# Beyond the Signal: Imaging Insights and Diagnostic Relevance of Bone Oedema in Bone Tumours and Tumour-like Lesions

**DOI:** 10.3390/cancers17132074

**Published:** 2025-06-20

**Authors:** Neel R. Raja, Hasaam Uldin, Ali Shah, Shashank Chapala, Rajesh Botchu

**Affiliations:** 1 Department of Radiology, Leicester Royal Infirmary, University Hospitals Leicester, Leicester LE1 5WW, UK; neel.raja@nhs.net; 2 Department of Musculoskeletal Radiology, Royal Orthopaedic Hospital, Birmingham B31 2AP, UK; hasaam.uldin1@nhs.net; 3 Department of Radiology, Nottingham University Teaching Hospital NHS Trust, Nottingham NG7 2GT, UK; ali.shah1@nhs.net; 4 Department of Radiology, AIG Hospital, Hyderabad 500032, India; chapalashashank@gmail.com

**Keywords:** magnetic resonance imaging, MRI, bone, Oedema, bone tumour

## Abstract

Bone oedema is associated with a wide spectrum of pathologies, including inflammation, infection, trauma, and neoplasms. The imaging characteristics of bone oedema in the context of an osseous tumour or tumour-like lesion can help narrow the aetiological differential and, when combined with specific MRI sequences or additional imaging modalities, may even suggest the precise underlying diagnosis. In this review, we discuss the various imaging modalities used in the detection and characterisation of bone oedema, briefly describe histopathological correlate of this finding, and detail the imaging features of a range of benign and malignant lesions that are classically associated with bone oedema. Finally, we explore the role of bone oedema in assessing treatment response, in relation to chemotherapy and radiotherapy.

## Simple Summary

Bone oedema, both within and on the surface of a bone, is a reactive process which occurs in a range of conditions, from benign to malignant. Using different imaging techniques, the volume, pattern and anatomic location of this oedema can be assessed. This can guide the radiologist and clinical team towards the underlying diagnosis, and therefore direct the most appropriate treatment for the patient. In this review, we discuss the different imaging techniques used in assessing bone oedema, their pros and cons, and imaging appearances of various conditions, with a focus on bone oedema. Finally, we describe how the pattern of bone oedema can provide information about how a patient’s cancer is responding to treatment.

## 1. Introduction

Bone oedema is a non-specific imaging finding associated with a wide range of pathologies, including trauma, infection, inflammation, and neoplasms [1,2]. Accurate detection of bone oedema is essential for diagnosing and managing a multitude of musculoskeletal conditions, in particular bone tumours and tumour-like lesions but also trauma and infection amongst others. Magnetic resonance imaging (MRI) is generally regarded as the optimal imaging modality for assessment of bone oedema related to this particular family of pathologies [3]. Through the utilisation of different pulse sequences, the extent and morphology of lesion-related oedema can be established, which offers numerous diagnostic advantages [4]. In certain cases, the MRI appearances of a lesion or process are so characteristic that the diagnosis can be made purely radiologically. However, in cases where imaging is not conclusive, MRI can still offer important information about the best site from which to obtain a biopsy, in order to increase the likelihood of definitive histological assessment [1,4].

In this review article, we will discuss imaging modalities which can demonstrate bone oedema, along with their respective strengths and weaknesses. We will also briefly detail the histological correlation of radiological bone oedema, as well as describing common bone tumours and tumour-like lesions which are commonly associated with osseous oedema, along with their imaging findings. We will conclude by describing the role of bone oedema in assessing treatment response, in relation to chemotherapy and radiotherapy.

## 2. Imaging Modalities for Oedema in Bone

As previously written, MRI is the primary modality utilised in the assessment of bone oedema, due to its high sensitivity for changes in water content and excellent soft tissue contrast. An imaging protocol to evaluate an osseous tumour or tumour-like lesion requires a multiplanar combination of different pulse sequences to exploit the differing physical properties of the constituent components of bone, along with the local soft tissues [2,4,5]. A typical protocol utilises a combination of proton density and T1-weighted and T2-weighted sequences, being sure to make use of fat suppression techniques, such as inversion recovery or fat saturation. Bone oedema appears as high signal in the all of these sequences, barring T1-weighted sequences, in which regions of oedema, usually within marrow fat, replace the usual high marrow signal with an intermediate to low signal intensity [6]. There are studies which demonstrate the ability of dynamic contrast-enhanced MRI to show bone marrow oedema, which is inferred by the increased rate of enhancement of the oedematous bone, as well as that immediately adjacent to it, accompanied by either an early plateau or washout [7,8]. While bone oedema may be conspicuous on unenhanced fluid-sensitive fat-suppressed MRI sequences, the real margin of a bone lesion can be better differentiated from perilesional oedema with the use of contrast [8].

Dual-energy computed tomography (DECT) has emerged as a promising alternative modality in the assessment of bone oedema, particularly in cases where MRI is contraindicated or unavailable. DECT makes use of the differential attenuation of X-ray photons of different energies, when they pass through a scan range containing various tissue types. Post-processing allows for virtual non-calcium images to be created, by subtracting the calcium content from bone, thereby highlighting areas of increased water, inferring the presence of bone oedema [9]. There are multiple studies which compare the sensitivity and specificity of DECT and MRI in the assessment of bone oedema. These report DECT as being less sensitive than MRI in bone oedema identification, particularly in the hyperacute post-traumatic phase, however state that DECT is able to accurately identify bone marrow oedema in its own right, with a sensitivity and specificity of 85% and 96% respectively [10,11,12,13]. These systematic review and meta-analysis results support the suggestion that DECT is a workable, time-efficient alternate imaging modality to assess for the presence of bone oedema, when MRI is unsuitable. However, there remains an associated ionising radiation dose with DECT, which is not present in MRI.

Other radiological techniques which have been explored in the role of bone oedema assessment include SPECT-CT, PET-CT and PET MRI. All of these techniques come under the umbrella of hybrid imaging, in which functional and anatomic imaging modalities are combined, for improved localisation and diagnostic accuracy. They require the administration of a preselected radiopharmaceutical, which accumulates at specific sites, varying depending on the particular clinical question. Whilst there are reports of these modalities demonstrating increased radiotracer uptake at sites of bone oedema already identified on MRI, their associated radiation dose and poor time- and cost-efficiency make them unlikely to be widely utilised in the routine setting [14,15,16,17].

## 3. Bone Marrow Oedema Histology

The non-specific MRI finding of bone oedema reflects an increased water content of the tissues, which resultantly display increased MR signal on fluid-sensitive sequences. Whilst this was originally hypothesised to be secondary to true local oedema, there are numerous studies which correlate the radiological finding of bone oedema and its corresponding histopathology. There are multiple reported histological findings relating to this entity, including interstitial oedema, focal haemorrhage, fibrosis, altered vascularity (either hyperaemic or congestive), inflammatory infiltrates and osteoclastic activity [18,19,20]. The broad range of reported histological findings solidifies the non-specific nature of MRI-demonstrated bone oedema, to the point where it has been referred to as oedema-like marrow signal intensity, in understanding of the fact that there is often more than just true oedema relating to the altered MR signal [18].

For continuity, the entity of ‘oedema-like marrow signal intensity’ will be encompassed by the term ‘bone oedema’ in the subsequent article sections. This latter term includes changes in the marrow as well as the cortex and periosteum.

## 4. Bone Oedema in Tumours and Tumour-like Lesions

The presence of bone oedema, its volume and morphology, and temporal evolution in relation to an osseous lesion are useful in narrowing the differential diagnosis and can provide guidance as to whether the lesion is benign or malignant, summarised in Table 1. A grading system described to qualify bone oedema in relation to bone lesions defines grade 1 as oedema present but smaller than the lesion size, grade 2 as oedema equivalent to the lesion size, and grade 3 as oedema greater than the lesion size [5]. The study in which this grading system was originally utilised demonstrated that with increasing volumes of bone oedema in relation to the size of the lesion, the likelihood of the lesion being benign increases; i.e., the majority of lesions with grade 1 bone oedema were malignant and those with grade 3 were more likely benign/non-neoplastic [1,2,5].

## 5. Benign and Tumour-like Lesions

### 5.1. Chondroblastoma

**Figure 1:**
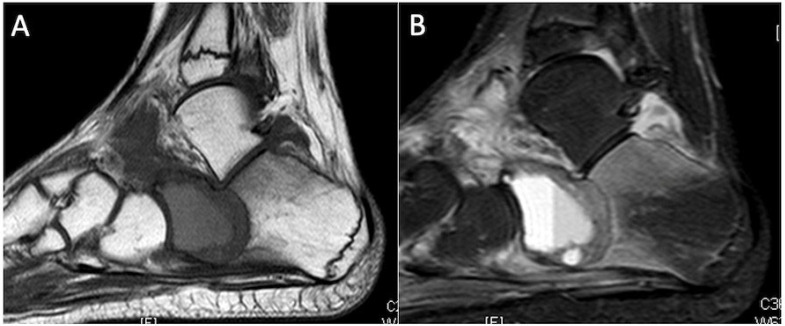
T1-weighted (**A**) and STIR (**B**) sagittal MRI demonstrating a chondroblastoma of the anterior calcaneus, with florid perilesional intramedullary oedema, greater in volume than the lesion itself.

Chondroblastomas are generally epiphyseal lesions, most commonly located in the distal femur, proximal tibia or proximal humerus, appearing as lucent with a sclerotic margin with or without central stippled calcifications [21]. On MRI, chondroblastomas demonstrate intermediate signal on T1-weighted and high signal on fluid-sensitive (e.g., STIR) sequences, with florid surrounding osseous oedema, predominantly intramedullary, which can extend to involve the metaphysis and even the diaphysis [22]. Moreover, there may also be associated soft tissue oedema, reported to occur in over 50% of chondroblastoma, relating to varying degrees of cortical breach (Figure 1) [23].

There are several radiological differentials for chondroblastoma, which include giant cell tumour of bone (GCTB), chondromyxoid fibroma, clear cell chondrosarcoma and a chondroblastoma-like variant of osteosarcoma [24]. GCTBs and clear cell chondrosarcomas, whilst both regarded as epiphyseal lesions, tend to arise between the third and fifth decades of life, in contrast to chondroblastomas, which are typically diagnosed in the second to third decades of life. Chondromyxoid fibromas are generally based in the metaphyses [24]. The volume of bone marrow and soft tissue oedema related to a chondroblastoma is greater than in clear cell chondrosarcoma [25]. Chondroblastoma-like osteosarcoma variants display different radiological features to chondroblastoma, in that they are ordinarily non-epiphyseal and demonstrate a greater degree of cortical destruction. However, the volume of oedema is similar between the two pathologies [26,27].

Despite these demographic and radiological differences, diagnosis can be challenging, and multidisciplinary team (MDT) involvement, including histopathology, is frequently utilised in order to plan the most appropriate management strategy. A description of the varying histological appearances of these differentials is beyond the scope of this article.

### 5.2. Osteoid Osteoma

Osteoid osteomas are benign bone tumours, typically affecting children in their second decade of life, with a classical history of night pain relieved by non-steroidal anti-inflammatory medication [28]. The radiological hallmark is that of a radiolucent nidus measuring up to 2 cm, best demonstrated by computed tomography (CT), usually surrounded by reactive sclerosis and periosteal reaction, which can be extensive enough to completely obscure the nidus [28]. The degree of reactive sclerosis and periosteal reaction varies depending on the nidus location, with cortical diaphyseal lesions demonstrating a greater degree of sclerosis than a subperiosteal lesion in either the metaphysis or epiphysis [29].

**Figure 2:**
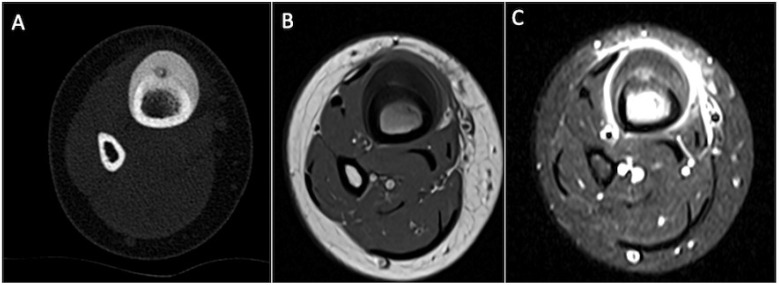
Lower leg axial CT image (**A**), axial T1-weighted (**B**), and STIR (**C**) axial MRI demonstrate the cortical thickening and extensive adjacent periosteal and soft tissue oedema.

Whilst thin-slice CT is the modality of choice for nidus identification, MRI characteristically shows marked bone oedema and adjacent soft tissue oedema (Figure 2). In cases of intra-articular lesions, joint effusions and synovitis are also present, also optimally demonstrated by MRI [30]. These MRI findings including the substantial oedema are valuable when an osteoid osteoma is located in a location where the degree of reactive bone formation is limited and the CT findings are therefore less pronounced, such as in the femoral neck and intramedullary portions of bone. The ‘half-moon sign’ has been described as a highly sensitive and specific finding on MRI for osteoid osteoma, referring to an area of bone marrow oedema in the femoral neck with a semilunar morphology, which can prompt targeted CT imaging to confirm the diagnosis and initiate timely treatment [31].

### 5.3. Osteoblastoma

**Figure 3:**
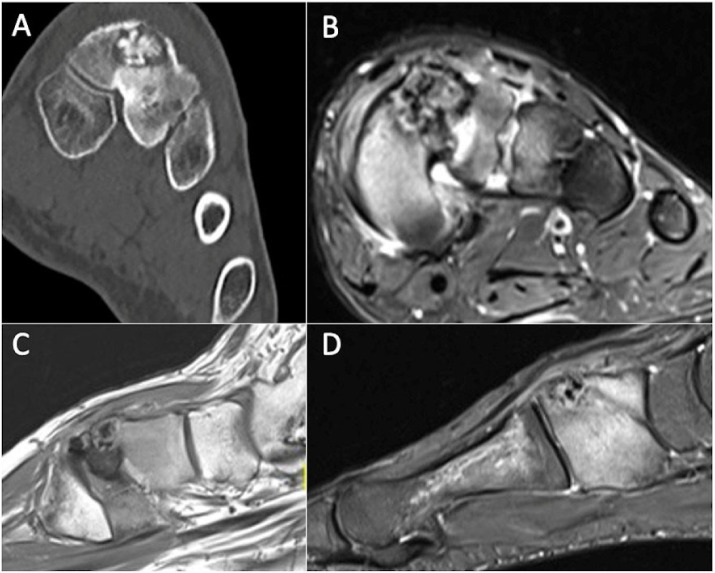
Coronal CT (**A**) and STIR MRI (**B**) demonstrating an osteoblastoma of the medial cuneiform, with marked surrounding sclerosis and both osseous and soft tissue oedema, larger than the size of the lesion itself. Sagittal T1-weighted (**C**) and STIR (**D**) coronal MRI demonstrate the oedema involving almost the entire medial cuneiform.

Osteoblastoma is a rare bone-forming tumour, accounting for less than 1% of all primary bone tumours, predominantly presenting in the second and third decades with a 2:1 male predominance [32]. The clinical presentation is comparable to that of the osteoid osteoma and these two entities are radiologically and histologically similar, with differentiation based on the size of the nidus: greater than 1.5–2 cm in osteoblastomas and smaller than this in osteoid osteomas [32]. Radiologically, these lesions can have non-specific appearances—lucent on radiographs and CT, with varying degrees of internal matrix calcification, and low to intermediate signal on T1-weighted and intermediate to high signal on T2-weighted sequences (Figure 3) [33]. Like osteoid osteomas, osteoblastomas can display marked oedema in the bone marrow and periosteum outside of the sclerotic tumour margins, as well as in the adjacent soft tissues [34].

### 5.4. Langerhans Cell Histiocytosis

**Figure 4:**
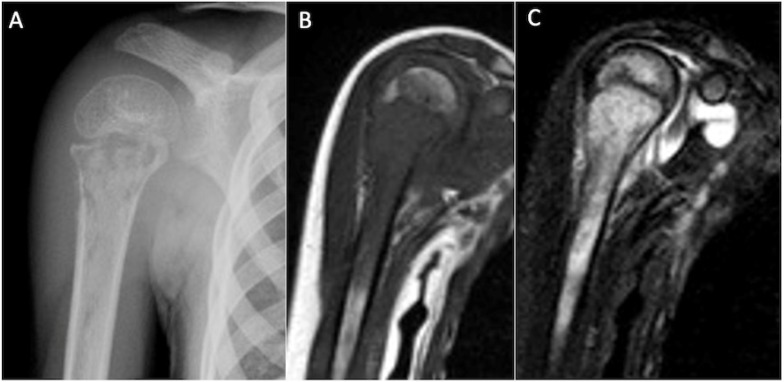
Anteroposterior radiograph (**A**) and coronal T1-weighted (**B**) and STIR (**C**) MRI showing Langerhans cell histiocytosis of the proximal humeral metadiaphysis, characterised by a permeative pattern of lysis and florid oedema throughout the affected medulla and adjacent soft tissues.

Langerhans cell histiocytosis is a disease of unknown aetiology, which, in the paediatric population, most frequently affects bone. There is infiltration and accumulation of abnormal histiocytes, preferentially involving the skull, ribs, pelvis and vertebrae [35]. Radiographically, these lesions appear most commonly as ill-defined, permeative lucent lesions, followed by lesions with destructive, expansile lytic and erosive morphologies (Figure 4) [36]. The utility of MRI in the assessment of these lesions cannot be understated, given that in some instances they only present radiographically once ~50% of the bone mineral density is lost [36]. On MRI, these lesions are of intermediate signal on T1-weighted and heterogeneously high signal on T2-weighted, and demonstrate avid enhancement on post-contrast sequences, along with extensive bone marrow oedema [35].

### 5.5. Stress Fracture

**Figure 5:**
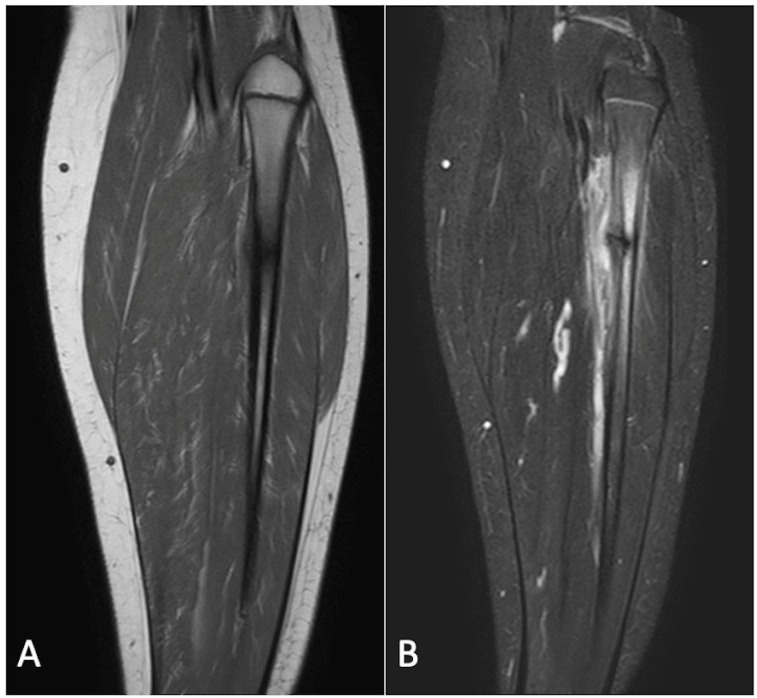
T1 (**A**) and STIR (**B**) coronal MRI images demonstrating stress fracture of the proximal fibula with intramedullary, periosteal and extraosseous oedema, along with a low signal fracture line.

Stress fractures are injuries which occur when the load placed on a bone is greater than its mechanical capacity. These can be subdivided into fatigue and insufficiency fractures, where there is abnormal force on normal bone, or normal force on abnormal bone respectively [37]. Radiographs are insensitive in the early stages of stress fractures, often resulting in MRI investigation due to patient symptoms, which demonstrates stepwise progressive osseous oedema, initially periosteal, then intramedullary, followed by a frank cortical fracture [38]. Despite the superior sensitivity of MRI, there is a minority of cases in which this stress response can be misinterpreted as tumour, given the low specificity of increased signal on fluid-sensitive and reduced marrow signal on T1-weighted sequences which can represent both entities (Figure 5) [39]. In such cases, correlation with the clinical history and anatomic location of the MRI abnormality is paramount, given that there are common sites for stress responses and fractures: femur, tibia, fibula, tarsals, metatarsals, pubic rami and sacrum [40]. Additionally, the focal thin-slice CT scan has been proposed as a useful investigation, due to its excellent spatial resolution and ability to demonstrate subtle fractures [39].

### 5.6. Infection

**Figure 6:**
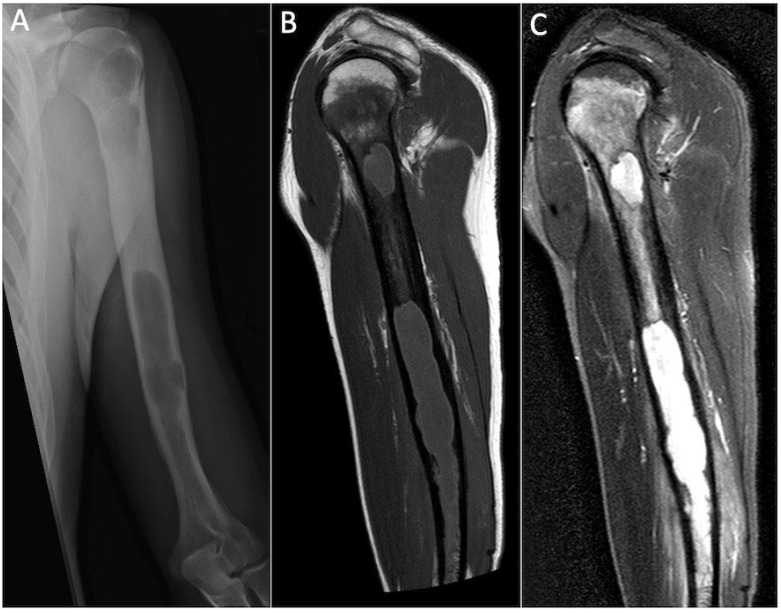
Humeral anteroposterior radiograph (**A**), sagittal T1-weighted (**B**) and STIR (**C**) MRI, demonstrating large intraosseous abscesses with the penumbra sign and marked surrounding intramedullary oedema, with free fat globules present, more so at the proximal metaphysis.

Osseous tumour and osseous infection are often indistinguishable clinically and radiologically, with the diagnosis being provided by culture and microscopy [41]. There are however imaging features which are sensitive and specific for osseous infection. MRI is very sensitive for the loss of the normal high marrow signal on T1-weighted imaging in early osteomyelitis, due to intramedullary oedema and exudates, which is generally of a much larger volume than the size of any focal abscess (type 3 bone oedema) [5,42]. More specific signs include the ‘penumbra sign’—which is demonstrated in subacute osteomyelitis, describing a rim of slightly higher T1 signal intensity at the periphery of a central osseous abscess compared with the abscess contents and the surrounding reactive oedema and new forming bone—and the presence of either intra- or extramedullary fat globules, which appear as high and low signal on T1-weighted and fat-suppressed sequences respectively [43,44]. Free fat globules are thought to reflect the result of infection-driven increased intramedullary pressure and subsequent adipocytic death, leading to their release. These globules are much larger than the foci of fat seen in cases of avascular necrosis, in which the morphology of the underlying adipocytes is preserved [44]. Identification of either the penumbra sign or free fat globules in a case of florid bone marrow oedema surrounding an apparent osseous tumour, would almost certainly reflect osteomyelitis, rather than a neoplastic process (Figure 6).

### 5.7. Periostitis Ossificans

**Figure 7:**
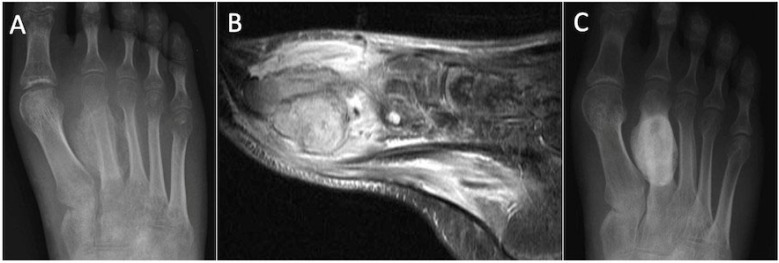
Dorsoplantar radiograph (**A**) and STIR sagittal MRI (**B**) demonstrating florid reactive periostitis and extensive local osseous and soft tissue oedema. Subsequent radiograph (**C**) demonstrates consolidation of the periostitis ossificans.

Periostitis ossificans is a rare benign surface lesion, characterised by hypertrophic ossification, with similar features to bizarre parosteal osteochondromatous proliferation, benign fibro-osseous pseudotumours, and turret exostoses [45]. The lesion demonstrates heterogeneously high signal on T2-weighted sequences with extensive bone marrow and soft tissue oedema, out of proportion to the degree of periosteal reaction on radiographs, which can be useful in distinguishing it from a malignant tumour (Figure 7) [45,46]. The main differential diagnosis is that of juxtacortical osteosarcoma. This latter entity is less common in the distal extremities but when it is located in more equivocal locations, such as long bones, histological assessment is required [46].

### 5.8. Calcific Tendinitis

**Figure 8:**
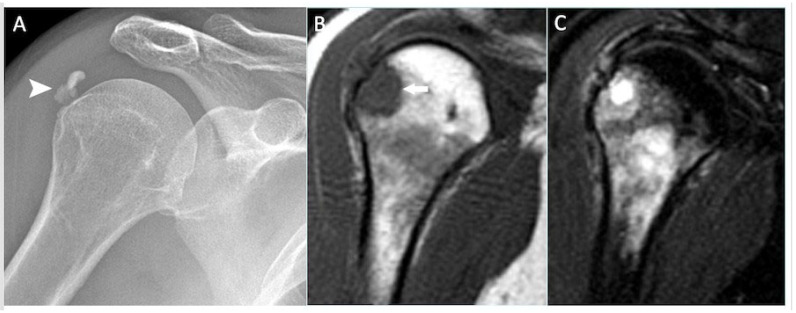
Shoulder anteroposterior radiograph (**A**), T1-weighted (**B**), and STIR coronal MRI (**C**) demonstrating calcific tendinitis of the supraspinatus tendon (arrowhead), with intraosseous migration (white arrow) and florid intramedullary oedema.

Calcific tendinitis, that is the deposition of calcium hydroxyapatite in tendons, is a common pathology which is better demonstrated radiographically or by CT than by MRI. Despite this, due to its prevalence, it is often seen on MRI during investigation for other pathologies, which can cause a diagnostic challenge [47]. During the resorptive calcific phase, there can be cortical erosion and intraosseous migration of the calcium, with florid osseous oedema demonstrated at affected tendon’s site of insertion (Figure 8). These imaging appearances can be mistaken for a more aggressive process, such as osseous infection or neoplasm. An acute clinical presentation, lack of joint effusion and absence of a soft tissue mass are key features favouring this as a diagnosis with localisation of the bone oedema to the site of tendinous insertion being a particularly helpful feature [48]. If not available, focused radiographs or CT should be performed to better assess for calcium hydroxyapatite deposition, which is self-limiting and can be managed conservatively based on patient symptoms [47].

### 5.9. Subtendinous Bone Oedema

**Figure 9:**
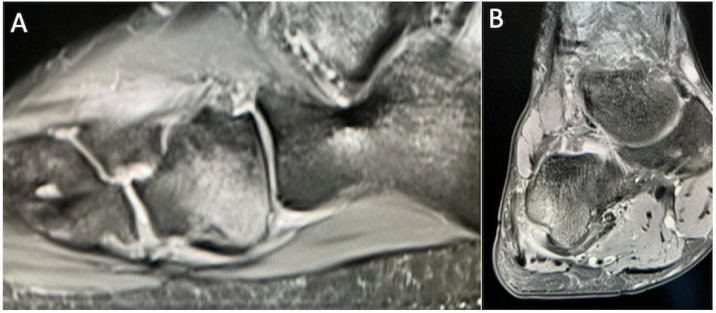
Sagittal (**A**) and coronal (**B**) STIR MRI demonstrating florid osseous oedema of the cuboid, secondary to peroneus longus tendinopathy.

Osseous oedema is frequently encountered in MRI of the foot and ankle, with a wide range of underlying aetiologies, including but not limited to trauma, neoplasm, infection and degeneration. Within this broad range, tendinosis is also a cause of bone marrow oedema, which occurs in a typically subtendinous distribution [49]. In severe cases, there can be associated erosion of the bone underlying the degenerating tendon, for example of the cuboid bone in relation to the peroneus longus tendon, in cases of cuboid tunnel syndrome (Figure 9) [50]. Awareness of this process is key to avoid unnecessary biopsy, with its associated risks and morbidity.

### 5.10. Gout

**Figure 10:**
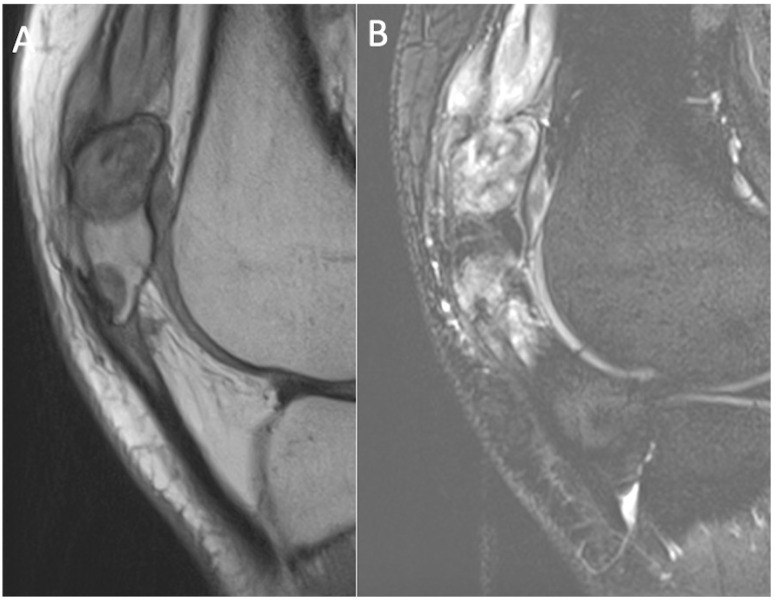
T1-weighted (**A**) and STIR (**B**) sagittal MRI demonstrating tophi at the distal quadriceps and proximal patellar tendons, with only mild oedema in the adjacent bone.

Bone oedema in gout is a controversial topic, regarded as mild and reactive to either an erosion or tophus in some literature, with florid bone marrow oedema reported to be present in gout complicated by concurrent osteomyelitis [51,52,53,54]. However, there is literature which describes the presence of more severe bone oedema in episodes of acute gout, without evidence of superadded infection [55,56]. In cases of suspected gout, we would recommend thorough clinical and biochemical correlation, given the lack of specificity of MRI findings, in particular the presence of bone oedema (Figure 10).

## 6. Malignant Lesions

### 6.1. Osteosarcoma

**Figure 11:**
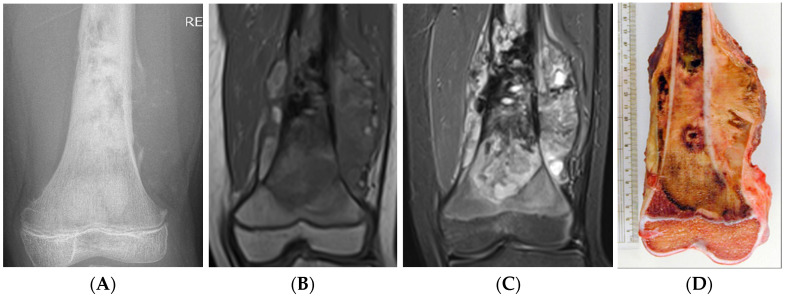
Anteroposterior radiograph (**A**) and T1-weighted (**B**) and STIR (**C**) MRI of a distal femoral osteosarcoma, demonstrating an aggressive periosteal reaction in the form of a Codman triangle, and a large soft tissue component. The volume of osseous oedema is less than that of the tumour itself. A macroscopic resection specimen of a different osteosarcoma is also shown (**D**).

MRI plays an essential role in the local staging of osteosarcoma, as well as identifying the relationship of the tumour to different muscle compartments and neurovascular bundles. T1-weighted sequences are of more use in defining the macroscopic extent of the lesion, when compared to fluid-sensitive fat-suppressed sequences which may overestimate the size of the lesion, due to their increased sensitivity for bone marrow oedema (Figure 11) [1,57]. The response to neoadjuvant chemotherapy can be assessed with conventional MRI, with a reduction in disease burden reportedly indicated through observing a decrease in perilesional oedema, the extent of marrow invasion and reduced tumour volume, as well as quantitatively by a reduced rate of contrast enhancement of the tumour in dynamic contrast-enhanced MRI [4]. There is also a potential role for diffusion-weighted imaging in assessing response, which if utilised early in the course of chemotherapy, could allow for treatment optimisation pre-surgery. A reduction in the apparent diffusion coefficient (ADC) is associated with a good response, reflecting tumoural necrosis and a modification in tumoural composition [58].

### 6.2. Chondrosarcoma

**Figure 12:**
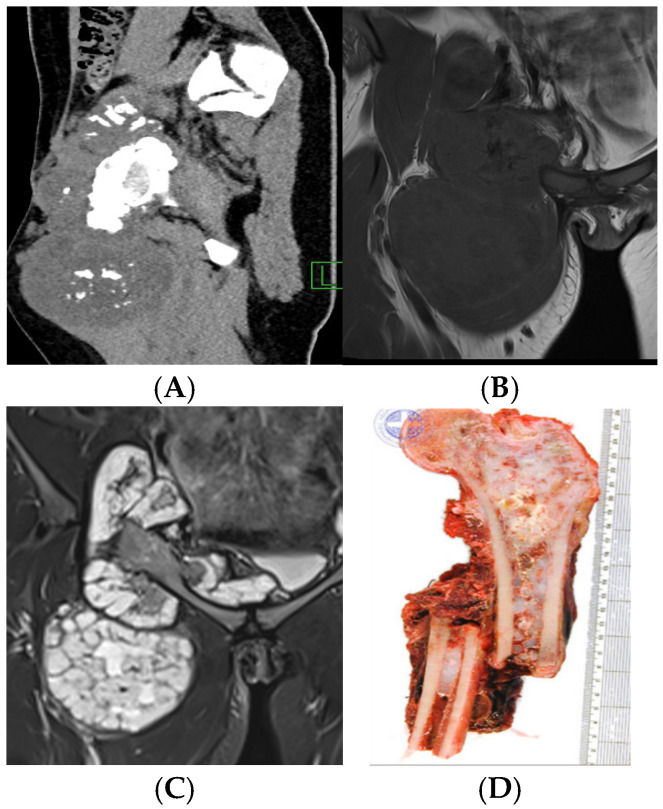
Sagittal CT reconstruction (**A**), coronal T1-weighted (**B**) and T2-weighted (**C**) MRI demonstrate a large soft tissue mass projected over the proximal medial thigh with internal calcification and no significant associated osseous or extraosseous oedema. Macroscopic resection specimen (**D**) of a proximal femoral chondrosarcoma, complicated by pathological fracture.

Chondrosarcoma is at the malignant end of the spectrum of central cartilaginous tumours, with the benign counterparts being enchondroma and atypical cartilaginous tumour, in ascending order of malignancy. Differentiation between enchondroma and atypical cartilaginous tumour is challenging both radiologically and histologically [59]. One study reported that the presence of perilesional oedema relating to a central cartilaginous tumour is suggestive of a chondrosarcoma, with this finding present in a greater proportion of high-grade lesions and not present in enchondroma [5,60]. Similar findings were reported in a more recent systematic review, and a suggestion was made that a combined assessment with MRI and radiography/CT would provide a more accurate assessment by incorporating an improved assessment of matrix mineralisation, endosteal scalloping and cortical breach as well as the oedema pattern (Figure 12) [61]. In a similar fashion to osteosarcoma, T1-weighted MR images are the most accurate sequences to define the size of a lesion, avoiding overestimation [57].

### 6.3. Ewing’s Sarcoma

**Figure 13:**
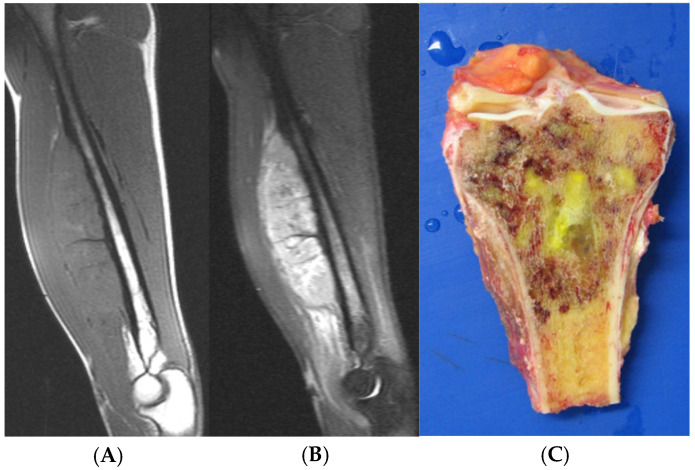
Sagittal T1-weighted (**A**) and STIR (**B**) MRI of a Ewing’s sarcoma of the humerus. Note the soft tissue component which is larger than the volume of the osseous oedema. Macroscopic resection specimen (**C**) of a proximal tibial Ewing’s sarcoma.

Ewing’s sarcoma is the second most common paediatric malignant bone tumour, with its peak incidence in the second decade of life and a significant proportion occurring in the third decade [62]. Both clinically and radiologically, Ewing’s sarcoma shares many features with osteomyelitis, making accurate differentiation challenging, but extremely important, given their entirely different treatment strategies. Patients may present with focal bone pain, fever and raised inflammatory markers, with radiographs of the affected bone demonstrating a permeative and cortical pattern of destruction, and an aggressive periosteal reaction, with or without articular involvement [63]. Bone oedema is present in both Ewing’s sarcoma and osteomyelitis, with the latter demonstrating a much greater degree in keeping with a trend of increased bone oedema generally favouring benignancy [5]. Additionally, one study demonstrated that a soft tissue mass demonstrated on imaging was significantly more suggestive of Ewing’s sarcoma than osteomyelitis (Figure 13) [64]. The soft tissue mass of Ewing’s sarcoma is more easily identified on fluid-sensitive sequences, when compared with T1-weighted, due to the similar signal intensity of extraosseous component and skeletal muscle on T1-weighted imaging.

## 7. Assessment of Bone Oedema Post Treatment

As detailed in the previous section, perilesional bone oedema is demonstrated in cases of neoplastic bone lesions due to surrounding neovascularity and inflammation. In cases of osseous sarcoma, neoadjuvant chemotherapy and/or radiotherapy are often utilised with the aim of reducing tumour bulk prior to surgery, but the interpretation of post-treatment MRI in the assessment of response has historically been challenging [65,66]. The importance of assessing for response to chemotherapy is that ineffective treatment can be terminated, thus avoiding the chemoradiotherapy side effects.

One study did not demonstrate a significant relationship between the volume of perilesional oedema and treatment response [66]. However, there are multiple studies which have shown that a reduction in the degree or resolution of peritumoural oedema on MRI is associated with a treatment response but also other studies showing that persistent or increased oedema indicates failure [67,68,69]. There are more recent studies which demonstrate the promising results of diffusion MRI sequences to quantitatively assess the tumour response after neoadjuvant chemotherapy, postulated to be due to a reduction in the cellularity of the tumour following treatment [70,71]. It is important to note, however, that these studies recommend the use of diffusion-based MRI sequences in addition to conventional qualitative sequences, rather than in isolation, and their conclusions were drawn from relatively small sample sizes.

## 8. Conclusions

There is a wide range of pathologies which can give rise to bone oedema, with tumours and tumour-like lesions comprising a significant proportion of them. Through the utilisation of fluid-sensitive and T1 MRI sequences, the presence of oedema can be sensitively identified and the macroscopic extent of a lesion can be determined accurately, respectively. These findings can both provide the MDT with useful information about the possible underlying aetiology as well as guide toward the most appropriate biopsy site where indicated.

To summarise, the volume of bone oedema in relation to a benign bone tumour is generally greater than the volume surrounding a malignant lesion. There are characteristic patterns of bone oedema in certain lesions, aiding specific diagnosis, however the presence of bone oedema associated with a tumour or tumour-like lesion is only a single piece of the puzzle. This must be combined with the remaining radiological, clinical and histological findings, in order to accurately diagnose and therefore manage the underlying process.

## Figures and Tables

**Table 1:** Summary of types of oedema present in, and MRI features of non-neoplastic and neoplastic pathologies.

Diagnosis	Volume of Oedema (Either Bone Marrow, Periosteal, Soft Tissue)	T1-Weighted Signal	T2-Weighted Signal	Extraosseous Component
Chondroblastoma	+++	Intermediate	High	Absent
Osteoid Osteoma	+++	Low	High	Absent
Osteoblastoma	+++	Low	High	Absent
Langerhans Cell Histiocytosis	+++	Intermediate	Heterogeneously high	Absent (present in extra-skeletal locations)
Stress Fracture	+++	Low	High	Absent
Infection	+++	Low, hyperintense rim ‘penumbra sign’	High	Present
Periostitis Ossificans	+++	Low to intermediate	Heterogeneously high	Absent
Calcific Tendinitis	+++	Low to intermediate	High	Absent
Gout	+ or +++ (if concurrent osteomyelitis)	Intermediate	Variable	Present
Osteosarcoma	+ or ++	Low to intermediate	Variable	Present
Chondrosarcoma	+ or ++	Intermediate to slightly high	Very high	Present
Ewing’s Sarcoma	+ or ++	Low to intermediate	Heterogeneously high	Present

+ oedema present but smaller than the lesion size; ++ oedema equivalent to the lesion size; +++ oedema greater than the lesion size.
